# An Object Feature-Based Recognition and Localization Method for Wolfberry

**DOI:** 10.3390/s25113365

**Published:** 2025-05-27

**Authors:** Renwei Wang, Dingzhong Tan, Xuerui Ju, Jianing Wang

**Affiliations:** College of Mechanical and Electrical Engineering, Harbin Engineering University, Harbin 150001, Chinaw18645568001@163.com (J.W.)

**Keywords:** wolfberry, harvest, robot, automatization, recognition, localization

## Abstract

To improve the object recognition and localization capabilities of wolfberry harvesting robots, this study introduces an object feature-based image segmentation algorithm designed for the segmentation and localization of wolfberry fruits and branches in unstructured lighting environments. Firstly, based on the *a*-channel of the Lab color space and the *I*-channel of the *YIQ* color space, a feature fusion algorithm combined with wavelet transformation is proposed to achieve pixel-level fusion of the two feature images, significantly enhancing the image segmentation effect. Experimental results show that this method achieved a 78% segmentation accuracy for wolfberry fruits in 500 test image samples under complex lighting and occlusion conditions, demonstrating good robustness. Secondly, addressing the issue of branch colors being similar to the background, a *K*-means clustering segmentation algorithm based on the Lab color space is proposed, combined with morphological processing and length filtering strategies, effectively achieving precise segmentation of branches and localization of gripping point coordinates. Experiments validated the high accuracy of the improved algorithm in branch localization. The results indicate that the algorithm proposed in this paper can effectively address illumination changes and occlusion issues in complex harvesting environments. Compared with traditional segmentation methods, it significantly improves the segmentation accuracy of wolfberry fruits and the localization accuracy of branches, providing technical support for the vision system of field-based wolfberry harvesting robots and offering theoretical basis and a practical reference for research on agricultural automated harvesting operations.

## 1. Introduction

Wolfberry, a significant economic crop in the Ningxia Hui Autonomous Region of China, is of significant importance for ecological and environmental protection, industrial structure optimization, increasing farmers’ income, and rural revitalization development [1,2,3,4]. Due to its rich nutritional and medicinal value, the demand for wolfberry continues to grow, and it has been widely used in food medicines, dietary supplements, and other related products [5,6,7], driving the demand for efficient wolfberry harvesting technologies. The epidermis of wolfberry fruits is relatively tender, tiny, and sensitive, making them susceptible to damage during harvesting [8,9,10]. Damaged fruits may turn black after drying, potentially reducing their economic value by up to 90%. Currently, wolfberry harvesting still relies on manual labor, which is not only inefficient but also costly, with labor costs accounting for over 40% of the total cost of wolfberry production. Replacing manual harvesting with robots can effectively alleviate the problem of labor shortages. This approach not only increases harvesting efficiency but also enhances harvesting quality and reduces production costs.

Machine vision is a key technology for wolfberry harvesting robots. Precise recognition and localization enable robots to accurately identify mature wolfberry fruits and precisely locate their positions, not only improving harvesting accuracy and efficiency but also reducing damage to plants, fruits, and flowers. Currently, the object recognition algorithms applied in agricultural harvesting robots mainly include object recognition and localization algorithms based on deep learning and image processing techniques.

Numerous scholars have conducted extensive research on the field of fruit object detection using deep learning technologies [11]. Song et al. [12] used the two-stage Faster R-CNN algorithm to detect kiwifruits under varying lighting conditions, achieving an accuracy of 87.61% and successfully capturing kiwifruits under different lighting scenarios. Liang et al. [13] utilized YOLOv3 to identify lychees at night and compared it with SSD and Faster R-CNN. Under low-light conditions, YOLOv3 demonstrated superior performance. Chen et al. [14] proposed an improved YOLOv3 algorithm for cherry tomato object recognition and localization, utilizing a dual-path network as a feature extractor to capture more detailed small object features, further enhancing the model’s detection capability for cherry tomatoes. The algorithm achieved an accuracy of 94.29%. This deep learning-based target detection technology in harvesting is not specifically for fruits mainly used in industrial processing. It is applied across various fruits and vegetables to boost harvesting efficiency, accuracy, and quality. While it can benefit subsequent industrial processing by providing better-quality raw materials, its focus is on optimizing the harvesting process in general. Qi et al. [15] used YOLOv5 to detect the main stems of lychees, achieving a recall rate and precision of 76.29% and 92.50%, respectively, for obtaining the 3D coordinates of the stem harvesting points. Although deep learning-based object detection technologies perform excellently in the accuracy of object recognition for harvesting robots, effectively training deep learning models requires a large number of image data samples and extended training time. Additionally, deep learning-based object detection technologies have limited adaptability to complex environments and are not suitable for object detection with small data samples. The complex environment of wolfberry recognition is mainly reflected in the variable fruit shape, shading by branches and leaves, uneven light, and external interference factors such as weather and terrain.

Traditional image processing techniques primarily rely on the color, shape, and texture features of the object, or on the fusion of multiple features to achieve object recognition and localization. Arefi et al. [16] first removed background information from the *RGB* color space, then integrated *RGB*, *HSI*, and *YIQ* color spaces to identify mature tomatoes. By utilizing the morphological features of images to accurately locate these mature tomatoes, Tanigaki et al. [17] segmented cherries from the background using color and spectral reflectance, and determined their positions using a 3D sensor. Malik et al. [18] used an improved *HSV* algorithm to segment tomatoes, and after morphological processing, employed the watershed algorithm to separate connected tomato fruits, achieving an accuracy of 81.6% in fruit detection. Li et al. [19] utilized the color characteristics of pineapples to segment them, and experiments showed that the recognition accuracy reached 90% in sunny conditions and 60% under cloudy conditions. Bulanon et al. [20] used the red color characteristic of apples and applied optimal threshold segmentation to separate apples from the background. This method achieved a success rate of over 80%, but in backlit environments, the error rate for apple recognition exceeded 18%. Zhou et al. [21] identified apples of different maturity levels based on the color difference *R-B* and *G-R* color models, with the correlation coefficients *R*^2^ between the detected apple count and manually counted apples, being 0.80 and 0.85, respectively. The *R*^2^ value between the number of detected apples and the actual harvest yield ranged from 0.57 after the fruits began to fall to 0.70 during the fruit maturation period. Segmentation based solely on color features is influenced by the maturity level of the fruits, the complexity of the background, and the intensity of the lighting. When the object to be recognized has a color similar to the background, shape features can be used to detect the object. For example, Whittaker et al. [22] proposed a tomato recognition method based on shape features, which can accurately identify tomatoes regardless of the maturity differences of the fruits. Hannan et al. [23] detected clusters of orange fruits using a perimeter-based detection method, evaluating recognition rates under different lighting conditions. In testing 110 photos, the algorithm successfully identified over 90% of the fruits. Kelman et al. [24] proposed an apple detection algorithm based on convexity testing, which analyzes edge intensity curves to determine if they conform to a three-dimensional convex shape, successfully identifying 94% of the apples. Fruits can be identified by their texture differences from background elements like leaves and branches. For instance, Zhang et al. [25] used Gabor filters for texture segmentation, Kurtulmus et al. [26] detected green citrus with circular Gabor textures, and Chaivivatrakul et al. [27] recognized pineapples and bitter gourds based on texture features. However, single-feature recognition has limitations. Feature fusion overcomes these. Song et al. [28] worked on apple segmentation, while Payne et al. [29,30] improved mango recognition algorithms.

While deep learning-based methods such as Faster R-CNN and YOLO variants achieve high accuracy in fruit detection tasks, they often require extensive annotated datasets and significant computational resources for both training and inference. These limitations make them less practical for real-time deployment on agricultural robots operating in unstructured field environments. Traditional image processing techniques, including color-based segmentation in *HSV*, *Lab*, and *YIQ* spaces, or texture-based approaches using Gabor filters, offer better interpretability and require fewer resources. However, many such methods are sensitive to lighting conditions and may struggle in scenes with complex occlusion or color similarity between objects and background.

Wang et al. [10] proposed an enhanced Yolov8n-Pose-LBD key point detection algorithm inspired by pose estimation. The model is improved by integrating LSKNet, replacing Upsample with Dysample, designing a small object detection layer, and adopting the WIoUv3 loss function. Experiments show that this model has fewer parameters, and its detection and positioning performance is superior to the original model, providing technical support for the intelligent picking of wolfberry. Additionally, image segmentation methods can effectively address the challenges of wolfberry fruits being occluded and clumped together through meticulously designed algorithms, demonstrating robust interference resistance. This study employs a fusion algorithm based on color space and clustering to segment and locate images of wolfberry fruits and branches to obtain the optimal harvesting positions, meeting the practical application requirements of harvesting robots, providing technical support for the vision systems of field-based wolfberry harvesting robots. The proposed method combines robust color features (from *a-* and *I*-channels) and wavelet-based fusion, achieving accurate segmentation without reliance on pre-training, extensive labeled data, or GPU acceleration. The specific contributions of this study are summarized as follows:

- A novel feature fusion algorithm is proposed to achieve pixel-level fusion of feature from different color spaces through wavelet transformation.
- A *K*-means clustering algorithm is proposed to accurately locate the grasping points on the branches based on the Lab color space.
- A coordinate prediction method is proposed for branch clamping points based on the position of the fruit with high applicability and robustness.

## 2. Materials and Methods

### 2.1. Image Collection

**Figure 1:**
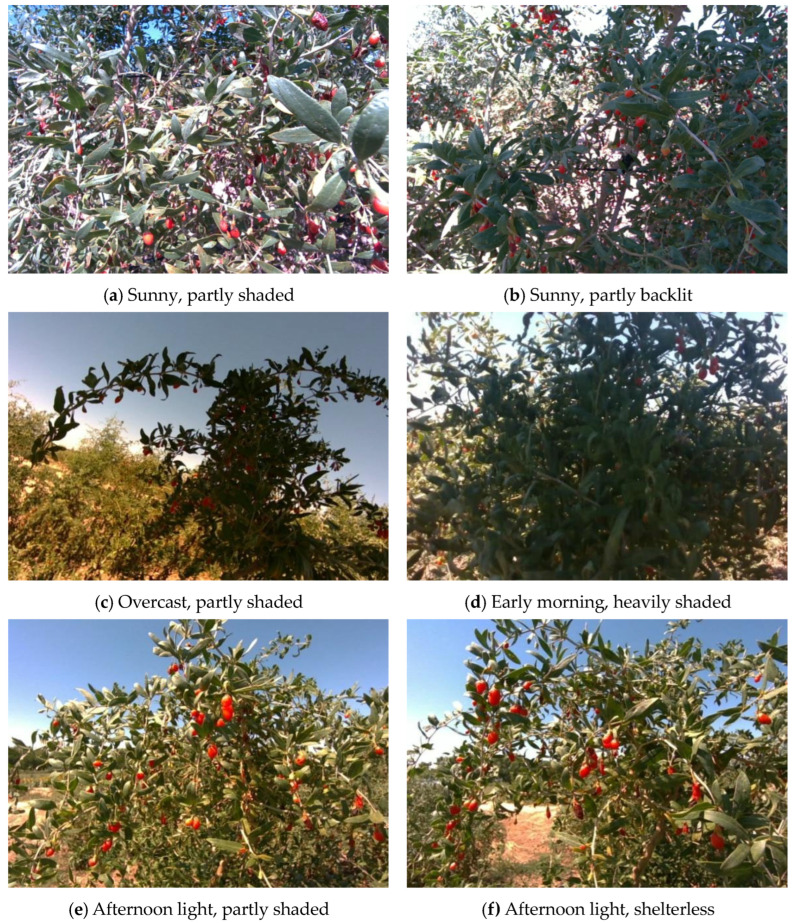
Wolfberry Fruit Images in Various Environments.

This study focuses on wolfberry trees located in Zhongwei City, Ningxia Hui Autonomous Region, China. The wolfberry trees are multi-branched shrubs of the Solanaceae genus. The color of wolfberry fruits changes significantly during the ripening process, gradually changing from green to bright red and eventually to deep red. The data samples were collected on 15 August 2023, using Intel RealSense D435i and D405 image acquisition devices (Intel, Santa Clara, CA, USA). To simulate the image capture conditions during robotic arm operations, the image capture distance was maintained within a range of 20 to 30 cm. After meticulous manual screening to remove low-quality samples, a total of 1200 images of mature wolfberries were ultimately collected. The collected images include wolfberry fruits under various lighting conditions such as sunny front lighting, sunny backlighting, side lighting, and cloudy days, encompassing complex scenes with severe occlusion and adhesion, as shown in Figure 1. All collected images were saved in PNG lossless compression format, with an image resolution set to 640 × 480 pixels.

### 2.2. Image Segmentation of Wolfberry Fruits Based on Feature Fusion

#### 2.2.1. Feature Image Extraction Based on Color Space Models

**Figure 2:**
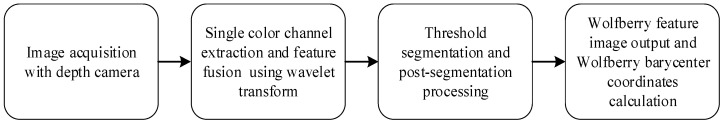
Feature fusion-based wolfberry fruit segmentation process.

*RGB* images and depth information of wolfberries are captured using a depth camera, and the RGB images are converted into Lab and *YIQ* color spaces. The a channel of the Lab color space and the *I*-channel of the *YIQ* color space are extracted as feature maps. Wavelet transform is then used, combined with a specific fusion strategy, to fuse the two-channel feature maps. Initial wolfberry fruit regions are extracted through threshold segmentation, and the segmentation results are further optimized using morphological processing and pixel area filtering, ultimately achieving precise segmentation of wolfberries. Unlike traditional pipelines that rely on pre-processing steps such as histogram equalization or color correction to address lighting variability, our method directly extracts robust color features from the *a*-channel of the Lab color space and the *I*-channel of the *YIQ* color space. These components are inherently less sensitive to illumination changes, reducing the dependency on explicit pre-processing. This design choice also enhances efficiency and simplifies deployment on embedded systems with limited computational capacity. Figure 2 shows the feature fusion-based process for wolfberry fruit segmentation.

This study selects the *a*- and *I*-channel images from the Lab and *YIQ* models for feature image fusion. To convert the original image from the *RGB* color space to the Lab color space, an intermediate *XYZ* color space is required. The *XYZ* color space can be obtained from the *RGB* color space through the following linear transformation Equation (1):

(1) $$\left[\begin{array}{c} X\\ Y\\ Z \end{array}\right]=\left[\begin{array}{ccc} 0.433953 & 0.376219 & 0.289828\\ 0.212671 & 0.715160 & 0.072169\\ 0.017758 & 0.109477 & 0.872765 \end{array}\right]\times \left[\begin{array}{c} r\\ g\\ b \end{array}\right]$$

In the Equation, *X*, *Y*, and *Z* represent the magnitudes of the three components in the *XYZ* color space; *r*, *g*, and *b* represent the normalized values of the three components in the *RGB* color space, which can be obtained from Equation (2).

(2) $$\left\{\begin{array}{c} r=R/\left(R+G+B\right)\\ g=G/\left(R+G+B\right)\\ b=B/\left(R+G+B\right) \end{array}\right.$$

The images directly obtained from the depth camera are in the *RGB* color space. After obtaining the three components of the *XYZ* color space, the three components of the Lab color space can be derived through Equations (3) and (4).

(3) $$\left\{\begin{array}{l} L^{*}=116\times f\left(Y\right)-16\\ a^{*}=500\times \left(f\left(X\right)-f\left(Y\right)\right)\\ b^{*}=200\times \left(f\left(Y\right)-f\left(Z\right)\right) \end{array}\right.$$

(4) $$f\left(t\right)=\left\{\begin{array}{ll} t^{1/3} & t>0.008856\\ 7.787\times t+16/116 & t\leq 0.008856 \end{array}\right.$$

The *a*-channel image displays *a* color transition from red to green, primarily reflecting red-green color variations and being less susceptible to illumination changes. Therefore, it can effectively distinguish mature wolfberries, green branches, and the background of immature wolfberries. This characteristic is also reflected in the grayscale distribution of the *a*-channel image. The grayscale histogram reflects the frequency distribution of each grayscale value.

The *YIQ* color space consists of three components: *Y*, *I*, and *Q*, where *Y* represents luminance information, *I* represents the color distribution from orange to cyan, and *Q* represents the color distribution from purple to yellow-green. The *YIQ* color space can be obtained through a linear transformation of the *RGB* color space, as shown in Equation (5):

(5) $$\left\{\begin{array}{c} Y=0.2990\times R+0.5870\times G+0.1140\times B\\ I=0.5957\times R-0.2745\times G-0.3213\times B\\ Q=0.2115\times R-0.5226\times G+0.3111\times B \end{array}\right.$$

In the equation, the *R*, *G*, and *B* values represent the three feature channels of the RGB image, while *Y*, *I*, and *Q* represent the three channels of the *YIQ* color space.

#### 2.2.2. Wavelet Transformation-Based Feature Image Fusion Algorithm

**Figure 3:**
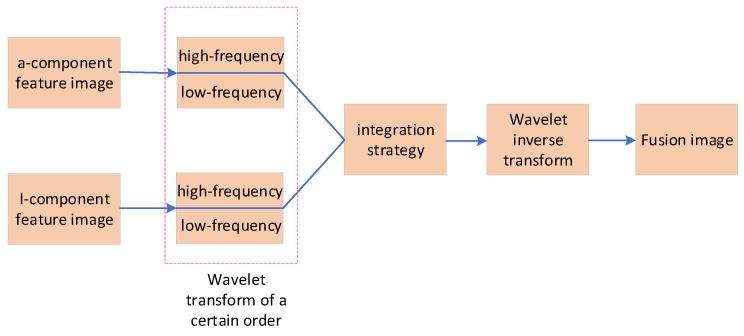
Feature Image Fusion Steps Based on Wavelet Transformation.

As shown in Figure 3, the wavelet transform-based image fusion method decomposes the images to be fused into sub-images containing high-frequency and low-frequency components. According to specific fusion strategies, these sub-images are fused to generate fused images at different scales, which are then reconstructed into the final image through wavelet inverse transformation. In the segmentation algorithm for wolfberry fruits, the a-component and *I*-component feature images are decomposed into sub-images containing high-frequency and low-frequency components. The high-frequency sub-image is fused with the low-frequency sub-image, and the fused image is obtained through wavelet inverse transformation.

The specific process of wavelet transform is as follows:

(1) Convert the *RGB* color space image into *LAB* and *YIQ* color space images.
(2) Extract the *a*-channel image (*a*-component feature image) from the LAB color space and the *I*-channel image (*I*-component feature image) from the *YIQ* color space.
(3) Convert the a-component feature image and the *I*-component feature image into double-type data.
(4) Set the wavelet decomposition level to 3, perform wavelet decomposition on the a-component feature image and the *I*-component feature image, and obtain the high-frequency and low-frequency parts.
(5) Fuse the high-frequency and low-frequency parts according to a specific fusion strategy, obtain multi-scale images, and reconstruct the fused wolfberry feature image using wavelet inverse transformation.

In wavelet transform-based image fusion, the higher the decomposition level, the better the fusion effect, but the longer the processing time. According to the existing research results [31], it can be seen that when the number of decomposition layers is 3, a better balance can usually be achieved in aspects such as detail extraction, computational load, and fusion effect, and therefore it is often the best. High-frequency sub-images reflect the image details, while low-frequency sub-images contain scale and contour information. Therefore, the fusion strategy is divided into high-frequency part fusion and low-frequency part fusion.

Since the grayscale values of wolfberry fruits are usually higher than the background, to better preserve features such as edges and textures of the wolfberry, the fusion of the high-frequency part uses the maximum absolute value method, i.e., the pixel value of the new image is taken as the one with the larger absolute value from the corresponding pixel of the original image. The fusion of the low-frequency part adopts a weighted average strategy, and the specific fusion coefficients are calculated using Equation (6).

(6) $$C_{f}\left(p\right)=\alpha \times C_{\alpha }\left(p\right)+\beta \times C_{l}\left(p\right)$$

In the formula: *C_f_*(*p*) represents the fused image; *C_a_*(*p*) represents the a-component feature image; *C_l_*(*p*) represents the I-component feature image; *α* represents the weight of the a-component feature image; *β* represents the weight of the *I*-component feature image. In this algorithm, both *α* and *β* are set to 0.5.

#### 2.2.3. Post-Processing of the Fused Image

For the segmented wolfberry fruit, the centroid of the region where the fruit is located represents the position of the wolfberry fruit. When calculating the centroids of different parts of the binary image, the black area is considered 0 and the white area is considered 1. The centroid coordinates of the wolfberry fruit are calculated using Equations (7) and (8).

(7) $$X=\frac{\sum_{j=0}^{N}\sum_{i=0}^{M}P\left(i,j\right)}{N}$$

(8) $$Y=\frac{\sum_{j=0}^{N}\sum_{i=0}^{M}P\left(i,j\right)}{M}$$

In the Equation, (*X*, *Y*) represents the centroid coordinates of the wolfberry fruit, where *M* and *N* are the number of rows and columns of the image, respectively. *P*(*i*, *j*) is the value of the pixel at the coordinates (*i*, *j*).

### 2.3. Clustering-Based Branch Recognition and Localization Method

#### 2.3.1. Clustering Segmentation Based on LAB Color Space

*K*-means clustering segmentation divides the image based on different colors, grouping regions with similar colors into one class. The *K*-means clustering algorithm follows these steps:

(1) Randomly select *k* points in the image as centers.
(2) Compute the distance of each sample point to these centers and assign the sample points to the class of the center closest to them.
(3) Recalculate the center of each class, using the mean of all sample features in that class as the center’s feature.
(4) Repeat steps (2) to (3) until the termination condition is met.

**Figure 4:**
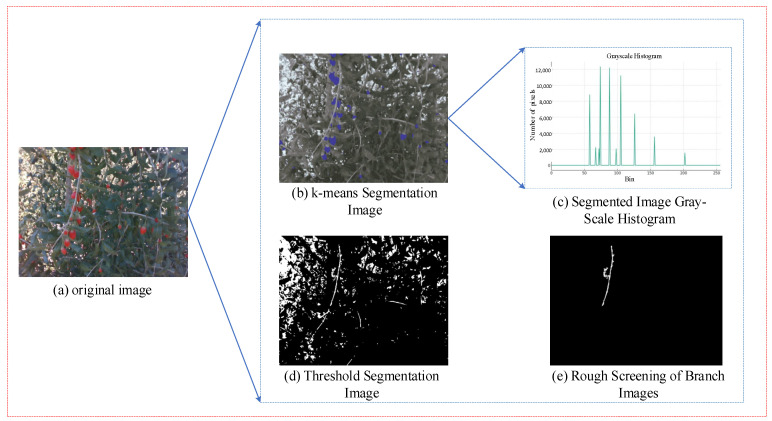
Post-processing of the wolfberry branch image segmentation.

The *K*-means clustering algorithm was selected due to its strong capability in grouping pixels based on color similarity, which is especially suitable for distinguishing wolfberry branches from fruits, leaves, and sky regions in the Lab color space. This approach is advantageous in scenes where illumination conditions vary significantly, as the color distribution remains a relatively stable feature. Through experimental comparisons across different values of *K* (ranging from 4 to 10), we observed that *K* = 8 consistently produced the clearest separation between branches and other background elements. While *K*-means alone cannot guarantee object-level accuracy due to noise and irregular shapes, the combination of morphological filtering and length-based post-processing allows for effective refinement of the segmented results, achieving accurate branch extraction under complex occlusion and lighting scenarios. The comparison before and after processing is shown in Figure 4b. After *k*-means clustering segmentation in the Lab color space, the branches, leaves, and wolfberry fruits are clearly distinguished by color. From the grayscale histogram (see Figure 4c), we can clearly observe that the image’s grayscale values are concentrated in several specific areas, forming distinct peaks. This distribution characteristic allows the histogram thresholding method to be effectively applied, and the segmentation result is shown in Figure 4d.

Compared to the wolfberry fruits, leaves, and sky regions, the branches are long, continuous areas, which can be selected by filtering the longest regions to remove non-object areas, thus obtaining the most complete branches in the image. The filtering process is as follows:

(1) Convert the uploaded image into a grayscale image and perform binarization.
(2) Find and analyze all contours in the image, identifying the longest contour by calculating the height of the bounding rectangle for each contour.
(3) Create a mask image that only covers the longest contour.
(4) Apply the mask to update the original image, thereby retaining only the highest region.

The filtered image after the process is shown in Figure 4e.

#### 2.3.2. Branch Clamping Point Localization

**Figure 5:**
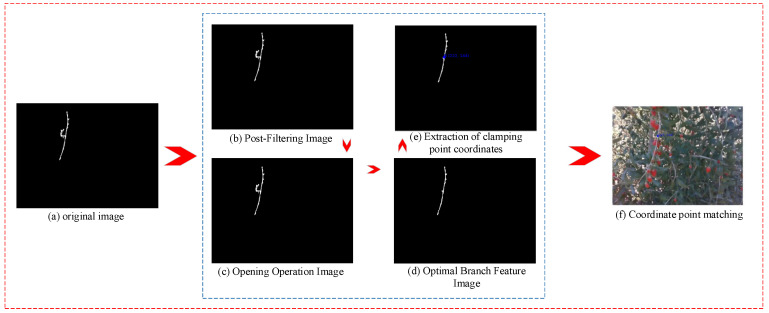
Branch Feature Point Calculation Process.

Median filtering is used to remove noise by replacing the value of a point with the median of the pixel values in its neighborhood, thereby reducing the influence of isolated noise points and making the pixel values closer to the true values. The current pixel and its neighboring pixels (such as a 3 × 3 or 5 × 5 window) are selected, sorted, and the median is taken as the current pixel value. A filter size of 3 is used for median filtering to remove noise. Figure 5b shows the comparison of the image before and after processing, with the noise in the original image effectively removed. After median filtering and denoising, the small noise points in the image have been removed. Next, morphological processing is applied to further remove burrs in the image to optimize the segmentation effect and improve image quality. To remove burrs in the image, morphological opening is used. The kernel size for the opening operation needs to be defined, and a kernel size of 2 × 2 is chosen in this paper. The processed image is shown in Figure 5c, where the burrs at the image edges are removed, the image becomes smoother, and the feature and non-feature regions are separated. After the opening operation, the longest region is selected to remove the non-feature regions. The final processed image is shown in Figure 5d, where the features of the branches are fully retained and non-feature regions are effectively removed.

After the branch segmentation is completed, a point on the branch needs to be selected as the gripping point for the robotic arm. The white area pixels are set to 1 and black area pixels to 0. First, the centroid of the segmented branch image is calculated, and it is determined whether the centroid lies within the branch area. If the centroid is on the branch, the centroid point is the gripping point; if the centroid is not on the branch, the point on the branch closest to the centroid is selected as the gripping point. The result of the gripping point calculation is shown in Figure 5e. To make the result more intuitive, the calculated gripping point coordinates are matched with the original image, and the gripping point location is marked in the original image. As shown in Figure 5f.

## 3. Results and Discussion

### 3.1. Feature Characteristic of Wolfberry Image

**Figure 6:**
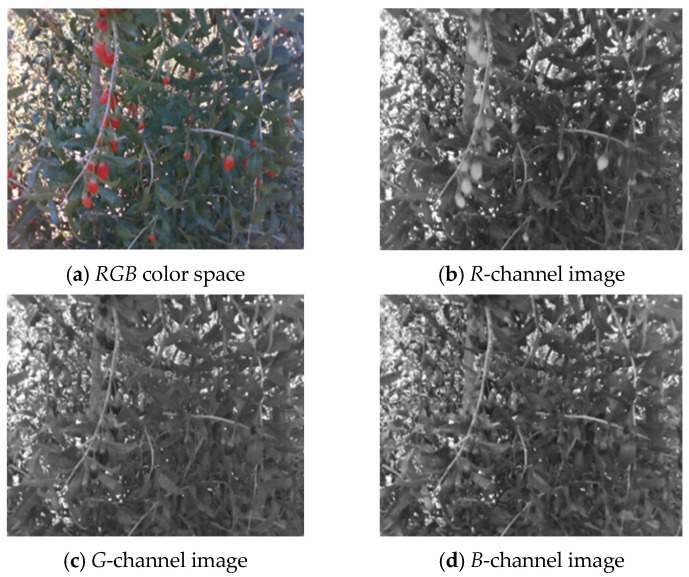
*RGB* color space wolfberry image.

**Figure 7:**
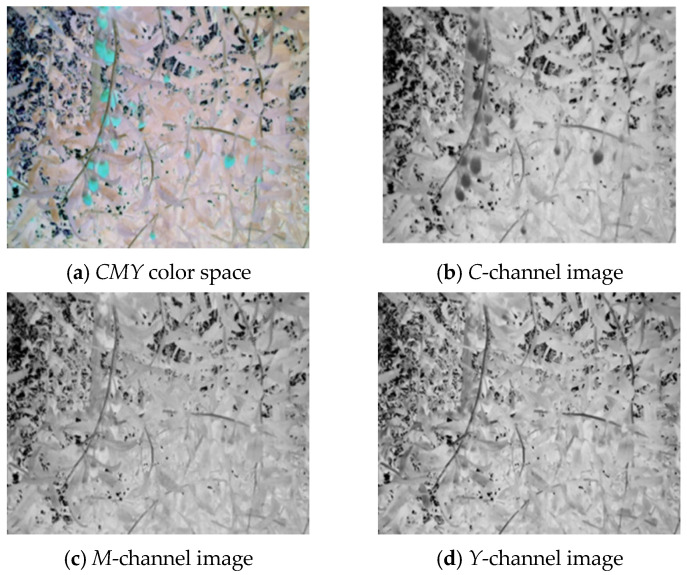
*CMY* color space wolfberry image.

**Figure 8:**
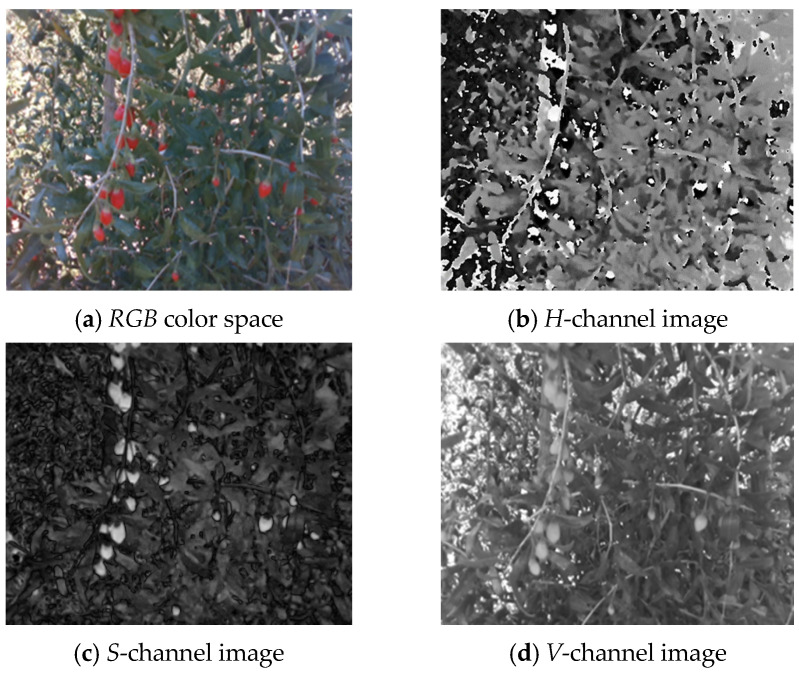
*HSV* color space wolfberry image.

**Figure 9:**
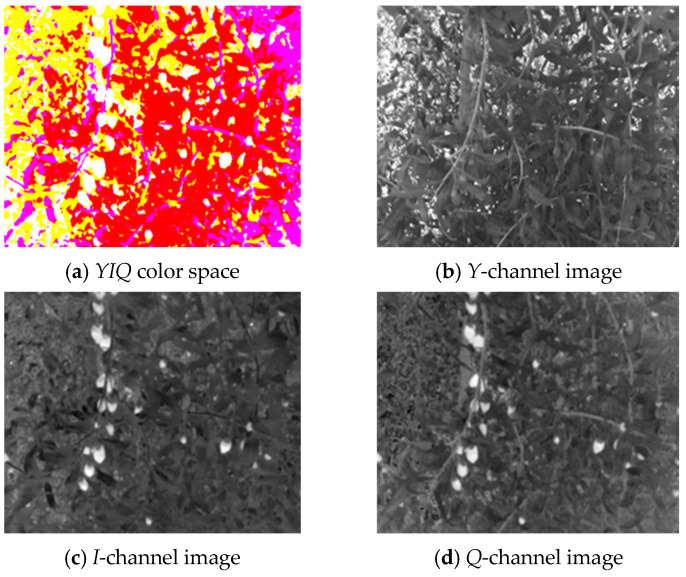
*YIQ* color space wolfberry image.

**Figure 10:**
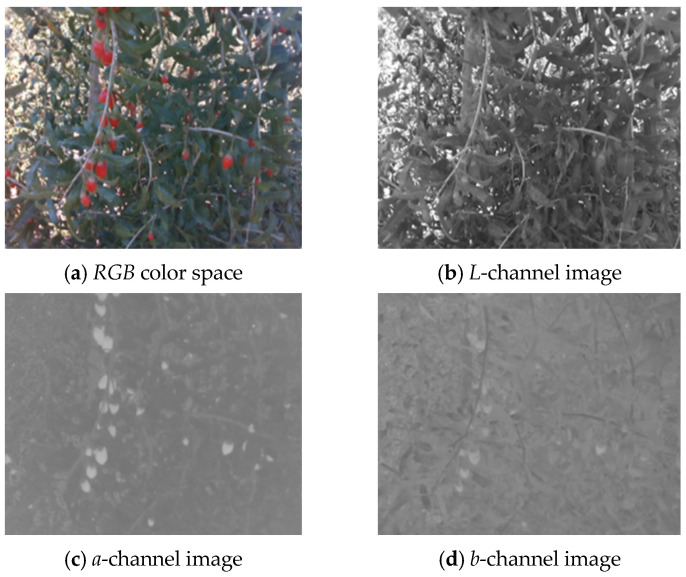
Lab color space wolfberry image.

Figure 6a–d respectively show the *RGB* wolfberry image captured by the camera, the *R*-channel image, the *G*-channel image, and the *B*-channel image. The *CMY* color space is composed of cyan, magenta, and yellow. Using white as the base color, different colors can be generated through the principle of color subtraction. Figure 7 displays the *CMY* color space image, the *C*-channel image, the *M*-channel image, and the *Y*-channel image, respectively. The *HSV* color space consists of Hue (*H*), Saturation (*S*), and Value (*V*). Hue represents color, Saturation reflects purity, and Value indicates brightness. Figure 8 shows the *RGB* image and its *H-*, *S-*, and *V*-channel images. In the *YIQ* color space, the *Y* component represents brightness, the *I* component represents hue, and the *Q* component represents saturation. Figure 9 shows the *YIQ* color space image, the *Y*-channel image, the *I*-channel image, and the *Q*-channel image, respectively. As shown in Figure 10, they are the original image, the *L*-channel image, the *a*-channel image, and the *b*-channel image, respectively. *L* represents luminance, a represents the distribution from green to red, and *b* represents the distribution from blue to yellow. Since the *a* and *b* components are independent of luminance *L*, external lighting changes do not affect their values. This characteristic allows the *a*-channel feature image to be used as one of the wolfberry feature images to be fused.

**Figure 11:**
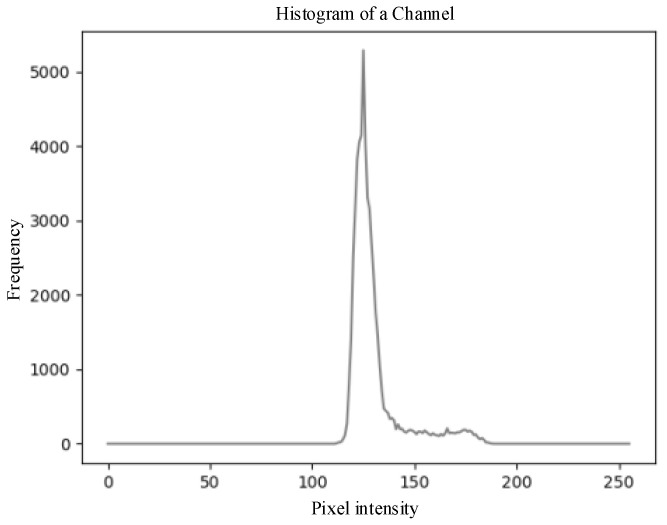
Grayscale histogram of the *a*-component feature image corresponding to the *RGB* image shown in Figure 10c.

As shown in Figure 11, the grayscale histogram of the *a*-channel exhibits a bimodal distribution, where the high peak at low grayscale values represents background pixels such as branches and leaves, and the low peak at high grayscale values corresponds to wolfberry fruits and some noise pixels. Due to the small gap between the two peaks and the low peak being easily overshadowed when the number of wolfberries is small, the low peak can become submerged.

**Figure 12:**
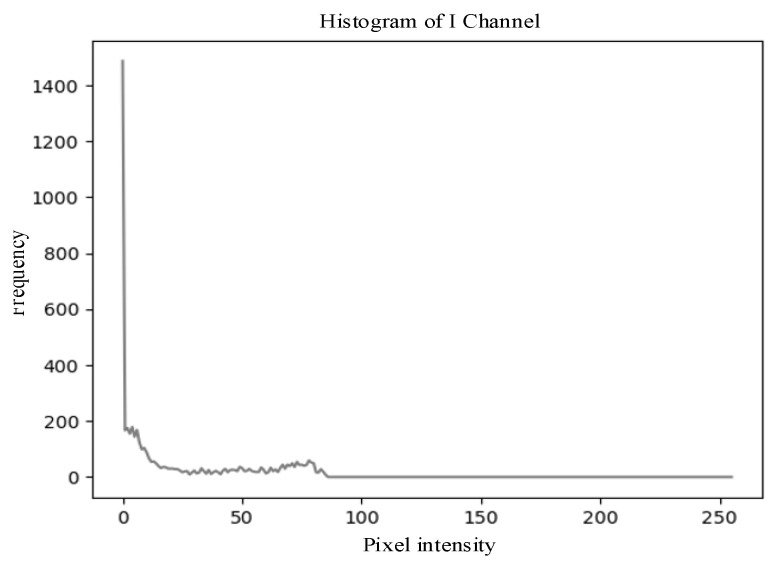
Grayscale histogram of the *I*-component feature image corresponding to the *RGB* image shown in Figure 9c.

From the aforementioned *I*-channel feature images, it can be observed that the color of wolfberries is relatively lighter compared to the environmental colors, thus enabling effective differentiation from the background. This can also be verified through the grayscale histogram of the *I*-channel. The grayscale histogram of the *I*-channel displays distinct distribution characteristics. As shown in Figure 12.

**Figure 13:**
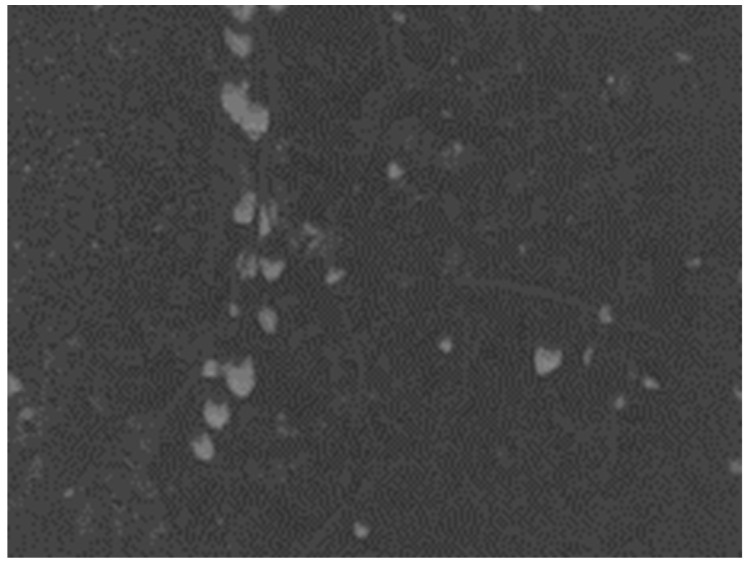
Wavelet transform fused wolfberry feature image.

The fused image is shown in Figure 13, successfully combining the advantages of the *a*-component feature image and the *I*-component feature image.

**Figure 14:**
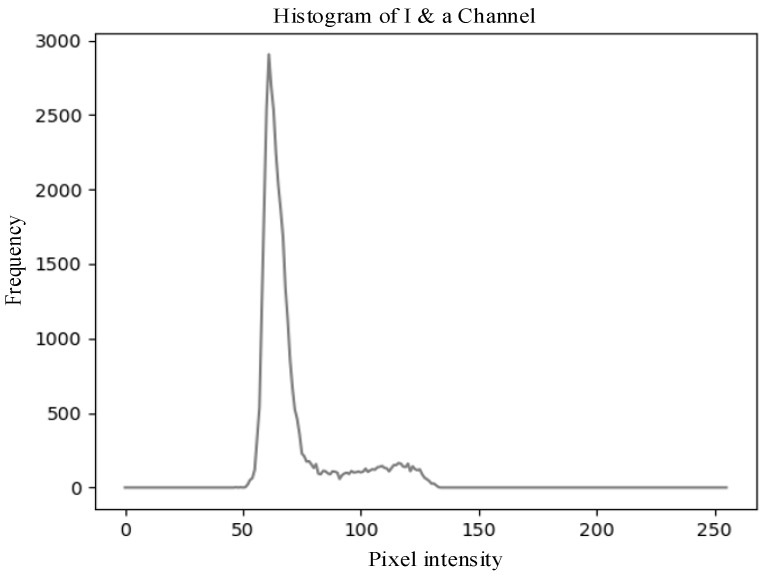
Grayscale histogram of the wolfberry fused image.

Figure 14 shows the grayscale histogram of the fused image generated by the fusion strategy. The histogram exhibits a bimodal distribution with a large gap between the two peaks. Histogram analysis shows that the fused image successfully combines the advantages of the grayscale distributions of the a-component and *I*-component feature images. After fusing the *a* component and the *I* component in Figure 14, the statistical characteristics show an essential difference compared with those of Figure 12. Although the two figures have similar appearances, Figure 14 can more comprehensively reflect the image details and structural information. The second peak, though low, contains more accurate texture distribution information. In subsequent applications, image enhancement and segmentation threshold selection can be optimized accordingly to improve the processing accuracy and robustness of the algorithm.

The selection of the *a*-channel (*Lab*) and *I*-channel (*YIQ*) was based on their ability to provide high contrast between target fruits and background foliage. This was verified through grayscale histogram analysis and preliminary segmentation tests, where these channels yielded the highest object separation clarity and accuracy. The segmentation correctness metric was used as the primary basis for assessing channel suitability.

**Figure 15:**
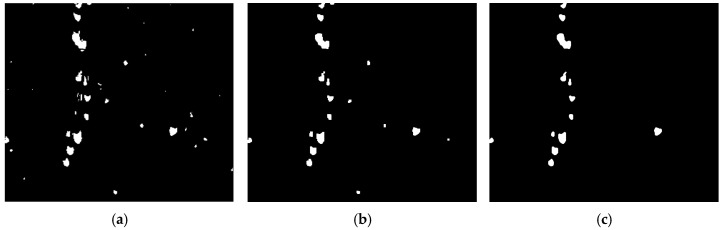
Morphological processing of fused wolfberry image: (**a**) Threshold-segmented wolfberry fusion image; (**b**) Wolfberry fusion image after opening operation; (**c**) Filtered wolfberry fusion image.

As shown in Figure 14, the grayscale histogram of the fused image presents two distinct peaks, with the lower peak corresponding to the wolfberry fruit and the higher peak representing the background. The threshold can be determined by identifying the valley between these peaks. The image segmented using the histogram technique is shown in Figure 15a. This study chooses to remove these noises through morphological processing. For the image after thresholding segmentation, the opening operation can remove small interference blocks. Figure 15b shows the image after the opening operation, where some tiny noise points have been removed. Then, the image after the opening operation is filtered to remove connected regions with an area of fewer than 100 pixels. Figure 15c shows the final processed image.

**Figure 16:**
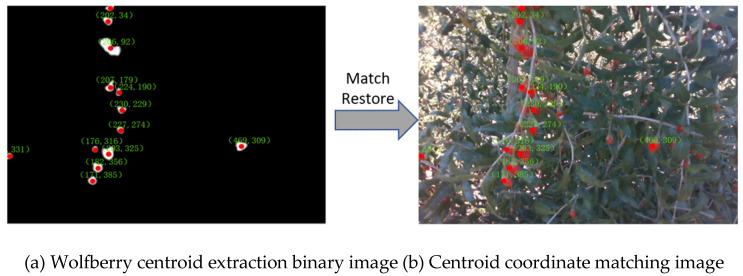
Wolfberry centroid acquisition and matching.

As shown in Figure 16a, the red marked points represent the centroid positions of the wolfberry fruit. For better visualization, the processed image is matched with the original image. As shown in Figure 16b, the pixel coordinates of the wolfberry are marked on the original image. By combining the depth camera and its relevant parameters, the pixel coordinates of these centroids can be converted into 3D coordinates, thereby achieving spatial positioning of the wolfberry fruit.

By observing the component images, it was found that the pixel differences between wolfberries and the background are significant in the *R*-, *H*-, *a*-, and *I*- channels, thereby making them suitable for object image segmentation. However, the *R*-channel is highly affected by illumination changes and is thus unsuitable for segmentation. The segmentation performance of the *I-* and *a*- channels is better, and the *I*-channel offers faster processing speeds.

### 3.2. Wolfberry Fruit Image Segmentation Experiment

Table 1 shows the grayscale distribution and threshold range of the single-channel feature image, as well as the grayscale distribution and threshold range of the fused feature image. As can be seen from the table, the histogram peaks of the fused feature image are more distinct, with a larger peak spacing, which significantly enhances the feasibility and accuracy of threshold segmentation.

Table 2 compares the segmentation accuracy of the wavelet transform-based feature fusion method with the segmentation accuracy of using only the *a*-channel feature image and the *I*-channel feature image. One representative image was used to manually tune the parameters (e.g., threshold values and morphological filters) of the image processing algorithms. These fixed parameters were then applied to the remaining 500 images to evaluate segmentation performance. As this study is based on rule-based image processing rather than machine learning, no iterative training or model fitting was involved. The image segmentation accuracy of the feature fusion method and the single-channel feature method was calculated respectively, with the formula for calculation shown in Equation (9). As can be seen from the table, the segmentation accuracy of the feature fusion method is better than that of the single-channel feature image, indicating that the feature fusion strategy significantly improves segmentation performance.

(9) $$Rate=\frac{\sum_{i=1}^{200}N_{i}}{\sum_{i=1}^{200}M_{i}}$$

In the Equation: *N_i_* is the number of wolfberries identified in the *i*-th image; *M_i_* is the actual number of wolfberries in the *i*-th image.

To provide a clearer evaluation of the real-time performance of the proposed methods, the average processing time per image was measured. On a standard PC equipped with an Intel Core i7-12700 CPU and 16 GB RAM, the wavelet transform-based feature fusion segmentation algorithm required approximately 0.63 s per image, and the branch localization algorithm based on K-means clustering required 0.41 s per image. These results indicate that the entire recognition and localization process can be executed in under 1.1 s per image on non-GPU hardware, making the method well-suited for resource-constrained field-deployed systems such as agricultural robots.

**Figure 17:**
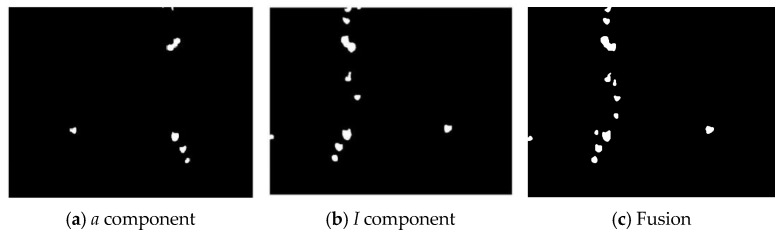
Comparison of segmentation experiments.

Figure 17 shows the experimental results of wolfberry fruit segmentation. It can be seen that the segmentation accuracy based on the a-channel and *I*-channel feature images is significantly lower than that based on the fused feature image. The feature fusion algorithm using wavelet transform effectively improved the segmentation accuracy of wolfberry fruit, validating the superiority of the feature fusion strategy in wolfberry recognition.

### 3.3. Wolfberry Branch Image Segmentation Experiment

**Figure 18:**
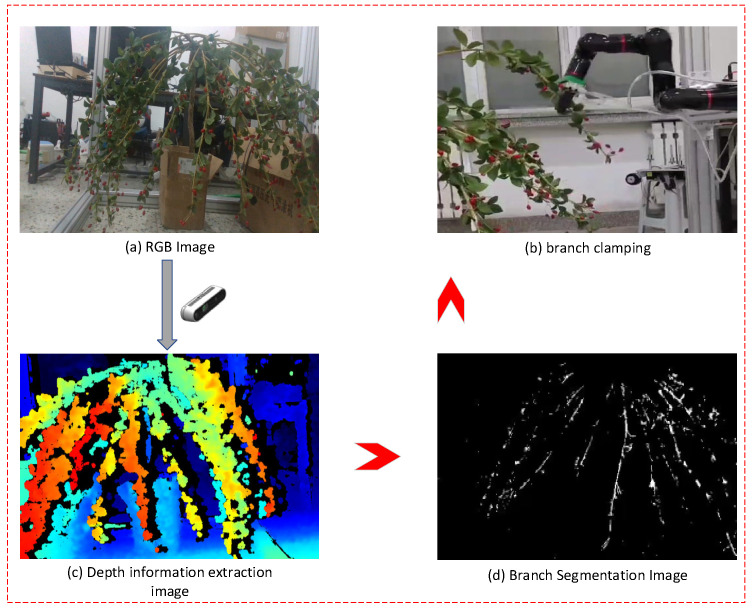
Field experiment image.

In this experiment, *RGB* images of wolfberry branches are first captured by a camera and converted from the *RGB* color space to the *Lab* color space. Then, the *K*-means algorithm is used for initial image segmentation. The segmented image is further processed by threshold segmentation to extract features, and the longest region is selected. The selected region is denoised using median filtering and optimized with morphological processing to improve image quality. Finally, length-based filtering is applied again to remove non-branch regions, achieving accurate branch identification. Figure 18a shows the *RGB* image captured by the depth camera, while Figure 18c is the corresponding depth image, where the grayscale value of each pixel represents the depth value of that point. After *K*-means clustering segmentation in the Lab color space and subsequent processing, the object branch is successfully segmented, as shown in Figure 18d. The longest branch is selected as the best object branch, and the centroid coordinates are calculated. The centroid is located on the branch, and the centroid coordinates correspond to the gripping point, with pixel coordinates of (350, 133) and a depth value of 144. The coordinates are converted to 3D coordinates and transmitted to the robotic arm. Figure 18b shows the experimental image of the branch being gripped. This study improves the object recognition and localization ability of wolfberry harvesting robots through a feature-based image segmentation algorithm. Our proposed wavelet transform-based feature fusion algorithm effectively improves the recognition rate of wolfberry fruit and reduces the impact of lighting and occlusion on recognition performance. Experimental results show that the recognition rate of the fused image segmentation algorithm is significantly improved, validating the potential of wavelet transform in feature fusion, especially its advantage in handling multi-scale features. This paper selects feature maps from the *Lab* and *YIQ* color spaces for fusion, emphasizing the importance of color space selection in improving segmentation algorithm performance, and provides new color space fusion strategies for future research. However, although the *RGB* and depth images were acquired using a calibrated RealSense camera system, no explicit pixel-level alignment between the *RGB* and depth frames was performed. As a result, there may be slight spatial deviations when mapping 2D image coordinates to 3D space. Addressing this limitation through precise *RGB*-depth registration will be considered in future work to further improve localization accuracy. In branch segmentation and gripping point localization, although the *K*-means clustering algorithm based on the *Lab* color space has achieved some success, there are still challenges in handling occluded branches, indicating the need for further research on more complex occlusion handling strategies to improve the robustness of the algorithm. Although both algorithms performed well in the experiments, there are still issues with sensitivity to image acquisition conditions, especially under lighting variations and fruit occlusion, and the real-time performance has not been fully validated in actual robotic arm operations. Future work will focus on algorithm optimization and integrated testing with actual robotic arms to improve practicality and robustness. This research provides a new perspective on object recognition and localization technology for wolfberry harvesting robots, promoting the advancement of agricultural automation, especially in wolfberry automation harvesting operations under complex field conditions, with significant practical implications. The team will continue to explore more efficient and robust image processing algorithms to meet the growing demand for agricultural automation.

## 4. Conclusions

The main conclusions of this study are as follows:

In this study, we proposed and validated a feature-based image segmentation algorithm that enhances the recognition and localization performance of wolfberry harvesting robots under unstructured lighting conditions. The method relies on pixel-level image fusion of the *a*-channel from the Lab color space and the *I*-channel from the *YIQ* color space using wavelet transform. This fusion strategy effectively preserves discriminative features of wolfberry fruits, enabling accurate segmentation under complex lighting and occlusion scenarios.

Compared to single-channel segmentation using the a component (57% accuracy) or the I component (73% accuracy), our fusion-based approach improved recognition accuracy to 78%, demonstrating a clear performance advantage. For branch localization, we proposed a *K*-means clustering algorithm in the Lab color space, combined with morphological filtering and a centroid-based gripping point localization strategy, achieving high accuracy in identifying graspable branches.

Nonetheless, the proposed method still has limitations in dealing with fully occluded branches and image misalignment between RGB and depth channels. In future work, we plan to incorporate pixel-level annotation datasets, enable comparisons with other segmentation methods, and perform real-time testing on embedded robotic platforms to further validate deployment performance. These steps will strengthen the generalizability and practical application of the proposed vision system in automated wolfberry harvesting.

## Figures and Tables

**Table 1:** Effect of wavelet transform image fusion on gray level histograms.

Image Type	Gray-Scale Distribution	Split Threshold Range
*a*-component Feature image	110–180	80–180
*I*-component Feature image	0–90	20–80
Fusion Image	50–130	70–130

**Table 2:** Correctness of different recognition methods.

Image Type	Segmentation Correctness
*a*-component Feature image	57%
*I*-component Feature image	73%
Fusion Image	78%

## Data Availability

Data will be made available on request.
